# Emulsive Liquid–Liquid Microextraction for the Determination of Phthalic Acid Esters in Environmental Water Samples

**DOI:** 10.3390/molecules29245908

**Published:** 2024-12-14

**Authors:** Xinyuan Bi, Chi Zhang, Xiaorong Xue, Shangjun Su, Zhiping Yang, Xu Jing, Qiang Zhang

**Affiliations:** 1 College of Resources and Environment, Shanxi Agricultural University, Taigu 030801, China; bixinyuan98@163.com (X.B.); z591374178@163.com (C.Z.); r1044381617@163.com (X.X.); sxsushj@126.com (S.S.); yzpsx0208@163.com (Z.Y.); 2 Institute of Eco-Environment and Industrial Technology, Shanxi Agricultural University, Taiyuan 030031, China; 3 College of Agricultural Economics and Management, Shanxi Agricultural University, Taiyuan 030006, China; 4 College of Food Science and Engineering, Shanxi Agricultural University, Taigu 030801, China; x.jing@vip.163.com

**Keywords:** emulsive liquid–liquid microextraction, fatty acid, demulsifier, phthalic acid esters, greenness evaluation

## Abstract

A convenient, rapid, and environmentally friendly method, emulsive liquid–liquid microextraction combined with high-performance liquid chromatography, was established to determine phthalic acid esters in tap, river, lake, and sea water. After the method’s optimization, we obtained the appropriate volume of the extractant and pure water, the number of strokes, the separation methods, the mass volume fraction of the demulsifier, the demulsifier volume, the sample volume, the salt amount, and the pH conditions. This method requires only 200 μL of heptanoic acid (fatty acid) as the extractant and 75 mg of sodium acetate as demulsifiers for fast microextraction and separation, respectively, avoiding the use of further equipment. Emulsive liquid–liquid microextraction offers substantial advantages over dispersive liquid–liquid microextraction by eliminating the need for toxic dispersants, thereby preventing any influences of dispersants on the partition coefficients. The linear range of detection ranged from 0.5 to 50 μg L^−1^, with a limit of detection of 0.2 μg L^−1^ and a limit of quantitation of 0.5 μg L^−1^. The recoveries ranged from 80.2% to 106.3%, and the relative standard deviations ranged between 0.5% and 6.7%. Five greenness metrics confirmed that this method is environmentally friendly and aligns with the principles of green analytical chemistry. The proposed method achieved a greenness score of 8.42, surpassing that of other methods as evaluated using the SPMS. The novel method may well be a valuable technique for determining phthalic acid esters in water samples.

## 1. Introduction

Phthalic acid esters (PAEs) consist of a benzene ring with two ester groups positioned in the ortho-configuration [1], which are used as plasticizers to improve the plasticity, durability, transparency, and strength of materials [2]. Every year, three million metric tons of phthalate derivatives are applied in various industrially manufactured products [3], such as food packaging, fertilizers, pesticides, detergents, and other industrial products. Dimethyl phthalate (DMP), diethyl phthalate (DEP), dipropyl phthalate (DPrP), dibutyl phthalate (DBP), and dipentyl phthalate (DPeP) are commonly used PAE compounds. Furthermore, the integration of PAEs into plastic products primarily relies on weak physical interactions, such as hydrogen bonding and van der Waals forces, rather than chemical bonds [4], which significantly contributes to their environmental release. Consequently, the widespread usage of PAEs has resulted in their effortless permeation into the environment through leaching, evaporation, and abrasion [5], resulting in the contamination of ecosystems. Studies show that PAEs harm human health [6], reducing sperm quality, causing infertility, disrupting the nervous system [7], and potentially increasing the risk of cancers, especially breast and liver cancer [8]. Indeed, PAEs could be widely detected in groundwater, river water, and seawater [9]; therefore, it is necessary to develop techniques for detecting PAEs in water samples.

As PAEs have inherently low solubility, their concentrations in environmental water samples are often trace-level, requiring sample pretreatment before chromatographic analysis. Liquid–liquid extraction (LLE) and solid-phase extraction (SPE) have been traditionally used as prevalent pretreatment techniques [10]. However, the methods are often time-consuming and require substantial amounts of organic solvents, posing limitations in terms of efficiency and environmental impact. To mitigate these inefficiencies, liquid-phase microextraction (LPME) and solid-phase microextraction (SPME) have emerged as innovative alternatives. LPME, a miniaturized version of LLE, utilizes mere microliters of solvent and hastens extraction speeds [11]. SPME minimizes the use of solid adsorbent but causes lags in LPME in terms of the extraction velocity. LPME boasts advantages such as simplicity of operation, shortened processing time, and cost-effectiveness. Nevertheless, its primary drawback lies in the utilization of toxic dispersants to facilitate the dispersion of the extractant within samples. Commonly used dispersants, including methanol, acetonitrile, and acetone, contribute to an increase in the overall toxic solvent load during the extraction process. Furthermore, the dissolution of these dispersants in samples diminishes the partition coefficient of analytes, thus impeding their efficient transfer to extractants.

Emulsive liquid–liquid microextraction (ELLME) was reported (in 2024) as a novel extraction technique that does not use toxic dispersants [12]. Its fundamental principle involves the mixing of an extractant with water to create a concentrated primary oil-in-water (O/W) emulsion; it is then rapidly injected into the sample, resulting in the formation of a diluted secondary O/W emulsion. This diluted emulsion, composed of the extractant and sample, facilitates full contact due to its large interfacial area, enabling the rapid and efficient extraction of analytes in samples [13]. Compared to previously reported LPME, ELLME based on O/W emulsions offers several advantages, including shorter operation time, milder extraction conditions, lower analyte losses, and the elimination of the need for additional toxic reagents or specialized equipment [14].

In LPME, the choice of extractant is paramount, as it directly affects both the extraction efficiency and the recovery of target analytes [15]. Fatty acids derived from natural resources are non-toxic or of low toxicity to organisms, have good biocompatibility, and can efficiently separate target analytes, aligning with the principles of green chemistry and sustainable development [16]. Fatty acids may be used as extractants, which avoids the use of traditional organic solvents and reduces environmental pollution. Heptanoic acid stands out as a promising choice, offering advantages, such as high extraction efficiency, environmental protection potential, selective extraction ability, and fast extraction kinetics [17]. Heptanoic acid can ionize to form hydrophilic anions and possesses the properties of an anionic surfactant, enabling it to extract hydrophobic analytes into its formed micelles, thereby significantly improving extraction efficiency [18]. This innovative emulsive microextraction mechanism based on heptanoic acid can accelerate the rate of analyte allocation between phases, ultimately facilitating efficient rapid extraction. The type and volume of the extractant [19], the separation technology utilized, the sample volume, the salt amount, and the pH can impact the extraction efficiency and enrichment factors [15].

Phase separation not only affects the extraction efficiency of analytes but also influences the simplicity of extraction operation and the degree of environmental pollution. Therefore, continuous development of phase separation methods is necessary in the application and development of LPME. Centrifugation, as the convention phase separation method, is often time-consuming and resource-intensive, prolonging the process and requiring extra equipment. Demulsifiers characterized by high surface activity and low surface tension are potentially powerful separation agents, enabling rapid demulsification and phase separation [20]. Sodium acetate is a biodegradable and cost-effective chemical, aligning with the principles of green chemistry. The hydroxyl part of the acetate ion forms a hydrogen bond with water molecules, creating a hydrophilic head, while the sodium ion behaves as a cation that can potentially aid in the transfer of target analytes from samples to the extractant [21], thereby potentially increasing the extraction efficiency of LPME processes.

The present work aims to develop a novel method to determine five PAEs (i.e., DMP, DEP, DPrP, DBP, and DPeP) in water samples by ELLME using fatty acids. Also, some variables (e.g., the extractant volume, pure water volume, number of strokes, separation methods, mass volume fraction of the demulsifier, demulsifier volume, sample volume, salt amount, and pH) will be tested to improve the recovery of PAEs. Finally, the applicability of the method will be performed by analyzing real tap, river, lake, and sea water samples.

## 2. Results and Discussion

### 2.1. Optimization of the Extraction Procedure

Tap water was used as the water sample in the subsequent optimization test, and a concentration of 5 μg L^−1^ was spiked into the blank matrix of tap water (Figure S1). The recovery of PAEs was affected by an array of ELLME factors, including the extractant volume, the pure water volume, the number of strokes, the separation methods, the mass volume fraction of the demulsifier, the demulsifier volume, the sample volume, the amount of sodium acetate, and the pH. To refine and maximize the recovery, optimization experiments were designed to optimize one variable at a time, performing each extraction condition in triplicates. The initial parameters were established as follows: 200 μL of extractant and 600 μL of pure water were used, and mixed 8 times. The sample volume was set to 7 mL, with 500 μL of a 15% (*w*/*v*) sodium acetate trihydrate solution added as the demulsifier. No additional salt was added, and the pH was left unadjusted.

The method for calculating the extraction recovery is outlined below:

$$\mathrm{Extraction}\;\mathrm{recovery}\;(\%)=\frac{C_{\mathrm{measured}}\;-\;C_{\mathrm{real}}}{C_{\mathrm{spiked}}}\times 100\%$$

*C*_measured_, *C*_real_, and *C*_spiked_ represent the measured concentration of target analytes in spiked samples, the concentration of target analytes in real samples, and the spiked concentration of target analytes, respectively.

#### 2.1.1. Optimization of the Extractant Volume

**Figure 1:**
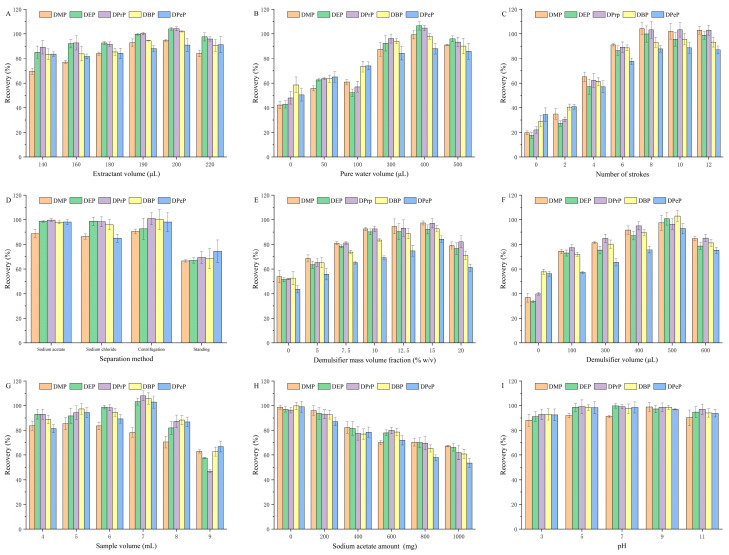
Optimization of ELLME parameters: (**A**) extractant volume; (**B**) pure water volume; (**C**) number of strokes; (**D**) separation method; (**E**) demulsifier mass volume fraction (% *w*/*v*); (**F**) demulsifier volume; (**G**) sample volume; (**H**) sodium acetate amount; (**I**) pH (the error bar represents the standard deviation).

The proper extractant volume is conducive to the separation of the extractant from the sample and ensures method sensitivity [19]. The effect of the extractant volume on the recovery range was studied with volumes ranging from 140 to 220 μL (Figure 1A). When the extractant volume increased from 140 to 200 μL, the recovery increased accordingly. This may be because an insufficient extractant cannot fully extract target analytes. When 200 μL was used, the recovery reached its peak and gradually decreased with further increases in the volume. It may be that excessive extractant affected the emulsion’s formation. Therefore, 200 μL of the extractant was chosen for further optimization in subsequent tests.

#### 2.1.2. Optimization of the Pure Water Volume

ELLME involves adding a concentrated O/W emulsion, which consists of heptanoic acid and pure water, to the sample, forming an emulsion system, and completing the extraction. The volume of pure water affects the emulsion concentration and recovery efficiency. The influence of the pure water volume on the recovery range was assessed with volumes ranging from 0 to 500 μL (Figure 1B). The best effect was achieved at 400 μL. An emulsion formulated with an optimal volume ratio may potentially enlarge the contact area between the extractant and analytes [13], thereby improving the recovery. When the volume of water was low, it was difficult for the emulsion system to form a stable O/W emulsion, and the inversion of the emulsion was more likely to occur. The high pure water volume led to low-concentration emulsion droplets. Therefore, 400 μL of the pure water volume was selected for further optimization in subsequent tests.

#### 2.1.3. Optimization of the Number of Strokes

The number of strokes affects the formation of the concentrated O/W emulsion and extraction efficiency. The effect of the number of strokes on the recovery range was studied with numbers ranging from 0 to 12 times (Figure 1C). The recovery increased with the increase in the number of strokes and remained unchanged after reaching eight strokes. It is likely that a certain number of strokes are required to sufficiently mix heptanoic acid and pure water, thereby ensuring the formation of the emulsion. Thus, eight cycles were selected for further optimization in subsequent tests.

#### 2.1.4. Optimization of Separation Methods

The choice of separation method can directly affect the extraction efficiency and time. Our study adopted the following separation techniques: adding organic salt (15% *w*/*v* sodium acetate) or inorganic salt (15% *w*/*v* sodium chloride) serving as demulsifiers, centrifugation (4000 rpm for 5 min), and standing for 30 min (Figure 1D). The recovery in sodium acetate and centrifugation treatment exceeds that of sodium chloride and standing treatment. The interfacial film at the O/W interface is more susceptible to disruption by external stimuli or droplet collisions, facilitating droplet agglomeration and enhancing the separation process [22]. Sodium acetate demonstrates remarkable surface activity and demulsification capacity, which can be used to exclude the use of a centrifuge and reduce the separation time. Its primary role is to diminish the free water surrounding extractant droplets via salt precipitation, thereby destabilizing the emulsion. Therefore, sodium acetate was selected as the demulsifier for further optimization.

#### 2.1.5. Optimization of the Mass Volume Fraction of Demulsifier

The mass volume fraction of the demulsifier determines its demulsification performance. The influence of the mass volume fraction of the demulsifier on the recovery range was estimated, ranging from 0 to 20% (Figure 1E). Within a certain range (0–15%), the stability of the emulsion system is generally enhanced with the increase in the mass volume fraction of the demulsifier. A moderate amount of sodium acetate can promote the breaking of the emulsion by altering the properties of the emulsion interface, such as reducing interfacial tension or increasing collision frequency. However, when the mass volume fraction of the demulsifier exceeds 15%, an excessive amount of demulsifier may increase the viscosity of the sample and alter the chemical environment of the emulsion, hindering interactions between the droplets. This is detrimental to breaking and subsequently to the emulsion, leading to a lower extraction efficiency. Therefore, 15% of the mass volume fraction was selected as the appropriate mass volume fraction of the demulsifier.

#### 2.1.6. Optimization of the Demulsifier Volume

As the volume of the demulsifier affects the phase separation time and efficiency, the influence of the demulsifier volume on the recovery range was studied across a range from 0 to 900 μL (Figure 1F). A large number of demulsifiers may be able to make fuller contact with the emulsion [23], thereby affecting the recovery. However, as the demulsifier volume exceeds a certain range, the interaction between the molecules of the demulsifier is enhanced, leading to heightened dissolution and consequently diminished demulsification effectiveness. The recovery was found to peak at 500 μL; thus, 500 μL of demulsifier was chosen as the optimal demulsifier volume.

#### 2.1.7. Optimization of the Sample Volume

The distribution coefficient of target analytes in the sample affects the mass transfer and then affects its recovery. The influence of the sample volume on the recovery range was investigated across a range from 4 to 9 mL (Figure 1G). The highest recovery was observed at 7 mL. Therefore, a sample volume of 7 mL was selected for the subsequent experiments.

#### 2.1.8. Optimization of the Salt Amount

To reduce the solubility in samples and improve extraction efficiency, adding a certain amount of salt to the sample may alter the partition between organic and water phases. The influence of the initial sodium acetate amount on the recovery was studied across a range from 0 to 1000 mg (Figure 1H). With the increase in the amount of initial salt added, the recovery decreased; this might be because excessive sodium acetate affected the partition coefficient between organic and water phases, thereby influencing the extraction efficiency and the recovery. Excessive amounts of sodium acetate may also increase the viscosity and density of the sample, reducing the extraction efficiency [24]. Therefore, additional sodium acetate was not added in subsequent experiments.

#### 2.1.9. Optimization of the pH of the Sample

The optimal pH value of the sample affects the analyte conversion into their neutral forms and the extraction efficiency [25]. The initial pH of the sample was 7.94; to investigate the effect of sample pH on the extraction efficiency, the pH values of samples were adjusted by 1 mol L^−1^ HCl and NaOH solution. pH values of 3, 5, 7, 9, and 11 were studied (Figure 1I). The extraction efficiency remained high at each pH value tested; thus, the experiment proceeded without any modification to the pH of the sample.

### 2.2. Validation Parameters of the Proposed Methodology

Tap, river, lake, and sea water were extracted using the optimal ELLME-HPLC method developed in this study. Mixed standard working solutions of various concentrations were prepared and spiked into the blank matrix of tap, river, lake, and sea water (Figure S1). The concentrations of the spiked standards were 0.5, 1, 5, 10, 50, and 100 μg L^−1^. Quantitation was performed using the external standard method. For each substrate (tap, river, lake, and sea water), the concentration of each PAE in the spiked samples was denoted by *x*, and the corresponding peak area obtained through detection was denoted by *y*. The linear equation, coefficient of determination (*R*^2^), limit of detection (LOD), limit of quantitation (LOQ), and intra-day and inter-day relative standard deviations (RSDs) for PAEs in each substrate were evaluated. The *R*^2^ values for all PAEs were greater than 0.998 (Table 1). The calculation of LODs and LOQs was based on a signal-to-noise ratio of 3 to 10 times. The LOD and LOQ were found to be 0.2 and 0.5 μg L^−1^, respectively. The intra-day (five times on the same day) and inter-day RSDs (five times on consecutive days) for each PAE ranged from 0.3% to 6.1% and from 1.1% to 9.1%, respectively.

To assess the suitability of the ELLME-HPLC method for detecting PAEs, initial tests on real tap, river, lake, and sea water samples were conducted. The results showed that these samples were free of detectable levels of PAEs. Subsequently, to further validate the method, spiked samples of tap, river, lake, and sea water with known concentrations of PAEs (0.5, 5, and 50 μg L^−1^, respectively) were spiked for analysis. As shown in Table 1, the recoveries obtained through standard addition ranged from 80.2% to 106.3%, and RSDs ranged from 0.5% to 6.7%. The results indicate that ELLME-HPLC is reliable and can be used to detect PAE residues in various water samples.

### 2.3. Evaluation of Eco-Profile

The Analytical Eco-scale (AES), the Green Analytical Procedure Index (GAPI), Analytical GREEnness (AGREE), the Analytical GREEnness metric for sample preparation (AGREEprep), and the sample preparation metric of sustainability (SPMS) were all considered when designing the methodology for evaluating greenness in this study.

AES as a semi-quantitative tool evaluates the environmental friendliness of an analytical method over its entire life cycle by considering factors such as the hazards posed by the reagents, the amount of reagents consumed, the energy consumption, and the amount of waste produced [26]. The evaluation is based on a maximum score of 100, with penalty points deducted for each criterion that does not meet ideal environmental standards. A higher score indicates a more environmentally friendly method. In general, a score of 75 or above is considered excellent green. In this instance, the method scored 85 (Table 2 and Table S1), suggesting that it is green.

GAPI also estimates the environmental friendliness of an analytical method by considering multiple aspects, including sample preparation, reagent usage, waste generation, and energy consumption [27]. This assessment tool uses pentagons, incorporating a color-coding system with three distinct hues, green, yellow, and red, to signify the level of environmental friendliness of the analytical technique. Upon evaluating the test method, it was found that seven aspects were rated green, six were rated yellow, and two were rated red (Table 2). Notably, both the amount of reagent used and the safety aspects were deemed green. Overall, the developed method was considered relatively environmentally friendly.

AGREE is a comprehensive evaluation method in green analytical chemistry that is specifically designed to quantify the greenness of sample preparation and analytical procedures [28]. It evaluates the environmental performance by considering green chemistry principles, including reagent dosage and toxicity, waste generation, energy requirements, the number of steps involved, miniaturization, and automation. Upon completion of the evaluation, the method received a final AGREE score of 0.68 (Table 2), indicating a relatively low negative environmental impact.

AGREEprep is an assessment tool grounded in green sample preparation (GSP) that is a methodology specifically designed to assess the greenness of sample preparation processes. Its primary objective is to assess the environmental impact of sample preparation methods. AGREEprep takes into account factors such as reagent dosage, waste generation, and energy efficiency during the sample preparation phase [29]. In this instance, 200 μL of heptanoic acid and 75 mg sodium acetate trihydrate were used in the process of sample preparation. Upon evaluation, the method received a final score of 0.71 (Table 2 and Table S2), indicating its high environmental friendliness and compliance with the GSP standard.

SPMS allows for a comprehensive examination of various factors involved in the sample preparation process, including resource consumption, energy usage, and pollutant emissions, thereby enabling a more precise assessment of its sustainability [30]. This methodology can help quantitatively analyze the sustainability of the sample preparation process, rendering the results more objective, detailed, and conducive to comparison. With an evaluation score of 8.42 (Table 2), the method shows a high level of environmental friendliness.

### 2.4. Comparison with Other Techniques

As shown in Table 3, the method proposed was compared with other liquid phase extraction methods for the chromatography analysis of PAEs, focusing on the analyte, environmental sample, pretreatment method, extraction reagent, extraction and separation device, detection instrument, LOQ, LOD, and ER. In the present study, five PAEs in four environmental water samples were detected. Only 200 μL of heptanoic acid and 75 mg of sodium acetate were used as these were considered green resources. In the reported cases, toxic and harmful reagents were used (e.g., 48 μL of dibutylamine, 200 mg of cresol, 20 μL of butyl lactate, and 400 μL of acetonitrile). Notably, the ELLME-HPLC method does not require the use of an ultrasonic bath, water bath, agitator, vortex mixer, centrifuge, or other equipment, which are typically required in the reported methods (e.g., ultrasonic bath for 10 min, water bath at 70 °C, vortex mixer for 1.8 min, centrifuge for 8 min, and agitator for 20 min). The LOQ and LOD are lower than the reported methods. The sample preparation metric of sustainability was adopted as a green evaluation methodology to evaluate the reported methods. The scores were 6.74 [31], 7.26 [32], 5.28 [33], and 5.58 [34], which are all lower than that of the proposed method, which was 8.42. In summary, ELLME has certain advantages in terms of greenness, simplicity, and sensitivity compared with other liquid phase extraction methods of PAEs.

## 3. Materials and Methods

### 3.1. Reagents, Chemicals, and Sample Preparation

DMP, DEP, DPrP, DBP, DPeP, heptanoic acid (98.0%), sodium acetate trihydrate (99.5%), sodium chloride (99.0%), acetonitrile (99.9%), and sodium hydroxide (99%) were obtained from Macklin Inc (Shanghai, China). Hydrochloric acid (37%) was obtained from Aladdin Co., Ltd., (Shanghai, China). Pure water was sourced from Barnstead™ GenPure™ Pro (Atvidaberg, Sweden). A stock solution of PAEs was prepared in acetonitrile at a concentration of 0.001g mL^−1^ and stored at 4 °C.

River and lake water were sourced from the Fenhe River and Jinyang Lake (Taiyuan, China), respectively. Sea water was sourced from the East China Sea (Xiamen, China), and the salinity was 29.8 g L^−1^. The samples were filtered through a 0.22 μm filter membrane.

### 3.2. Instrumental

Chromatographic separations were conducted using an ACQUITY Arc HPLC system with a Waters 2489 UV/Vis detector. A Waters XBridge C_18_ column (4.6 × 250 mm, 5 μm) was employed at a temperature of 30 °C. The mobile phase was a mixture of acetonitrile and pure water, with an initial composition of 60:40 (*v*/*v*) and a final composition of 80:20 (*v*/*v*) at 10 min and a flow rate of 0.8 mL min^−1^. The retention times of DMP, DEP, DPrP, DBP, and DPeP were 4.1, 5.6, 8.9, 13.4, and 18.8 min (Figure S1), respectively, with a wavelength set at 245 nm for detection.

### 3.3. ELLME-HPLC Protocol

**Figure 2:**
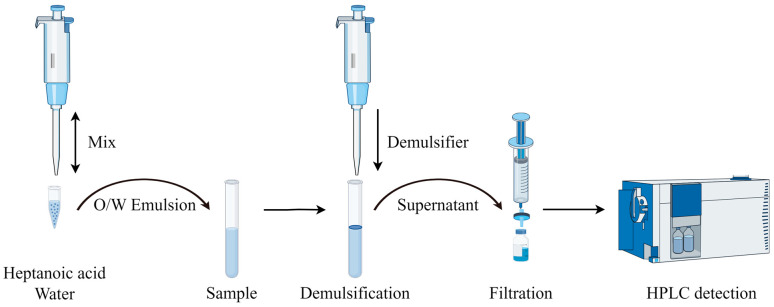
The flowchart of the ELLME-HPLC-UV/Vis by Figdraw 2.0 software.

At first, 200 μL of heptanoic acid and 400 μL of pure water in a 1.5 mL centrifugal tube were mixed eight times in 8 s with a plastic pipette tip to form a concentrated O/W emulsion. Then, the prepared emulsion was added to a glass centrifugal tube with 7 mL of a water sample to prepare a diluted O/W emulsion to complete the extraction. Finally, 500 μL of 15% sodium acetate solution as a demulsifier was added to fulfill phase separation within 2 min, and heptanoic acid was collected for analysis (Figure 2).

## 4. Conclusions

In this study, a simple, rapid, and environmentally friendly, method was developed to determine the amount of PAEs in tap, river, lake, and sea water. The renewable and environmentally friendly heptanoic acid was used as an extractant. It was mixed with pure water to prepare a concentrated O/W emulsion and then added to the sample to form a diluted O/W emulsion to complete the fast extraction. The organic salt sodium acetate was then added as a demulsifier to complete rapid phase separation (instead of using a centrifuge). The main advantages include the avoidance of toxic extraction solvents, operational simplicity, cost-effectiveness, and the elimination of the need for additional auxiliary equipment. Evaluation using AES, GAPI, AGREE, AGREEprep, and SPMS showed that the method aligns with the development principles of green analytical chemistry and demonstrates environmental friendliness. The proposed method utilizes 200 μL of heptanoic acid and 400 μL of pure water, which are mixed with eight strokes. Subsequently, 500 μL of a 15% (*w*/*v*) sodium acetate solution is added as a demulsifier, enabling the processing of a 7 mL water sample without the need for additional salt or pH adjustment. In conclusion, this method holds significant potential as an effective tool for the sustainable green extraction and chromatographic determination of PAEs in environmental water samples.

## Figures and Tables

**Table 1:** Assessment parameters of the proposed technique for detecting PAEs.

PAE	Sample	Linear Equation (μg L^−1^)	*R* ^2^	LOD (μg L^−1^)	LOQ (μg L^−1^)	Intra-Day RSD (%)	Inter-Day RSD (%)	Extraction Recovery		
								0.5 μg L^−1^	5 μg L^−1^	50 μg L^−1^
DMP	Tap	*y* = 25187.02*x* + 4026.76	0.999	0.2	0.5	4.4	4.7	91.8 ± 3.5	80.2 ± 2.5	83.1 ± 2.7
	River	*y* = 25697.58*x* + 7882.46	0.998	0.2	0.5	3.9	7.4	96.4 ± 4.2	87.2 ± 1.8	86.2 ± 1.3
	Lake	*y* = 25748.44*x* + 4358.51	0.999	0.2	0.5	2.8	1.8	88.0 ± 3.3	84.2 ± 2.1	83.2 ± 0.6
	Sea	*y* = 24738.13*x* + 4654.33	0.999	0.2	0.5	2.7	8.6	81.3 ± 2.1	81.5 ± 2.9	83.3 ± 1.6
DEP	Tap	*y* = 20798.26*x* − 2624.04	0.999	0.2	0.5	6.1	6.5	97.9 ± 2.6	89.9 ± 1.6	96.5 ± 2.2
	River	*y* = 21377.78*x* + 6516.51	0.998	0.2	0.5	1.5	6.9	99.4 ± 4.3	95.3 ± 5.4	99.3 ± 1.7
	Lake	*y* = 21329.38*x* + 6771.08	0.999	0.2	0.5	3.6	2.1	94.8 ± 4.1	95.3 ± 1.4	98.6 ± 3.1
	Sea	*y* = 20415.78*x* + 5976.21	0.999	0.2	0.5	2.3	2.4	103.9 ± 3.0	84.7 ± 5.3	99.9 ± 1.7
DPrP	Tap	*y* = 17303.09*x* − 6085.57	0.999	0.2	0.5	5.0	9.1	102.8 ± 1.6	91.0 ± 0.6	98.3 ± 1.7
	River	*y* = 17882.65*x* − 555.45	0.999	0.2	0.5	2.1	6.0	97.6 ± 4.6	95.6 ± 3.6	102.3 ± 0.8
	Lake	*y* = 17758.35*x* + 2071.82	0.999	0.2	0.5	1.4	1.1	92.8 ± 4.5	97.1 ± 2.2	100.0 ± 3.2
	Sea	*y* = 17029.26*x* − 307.17	0.999	0.2	0.5	3.4	6.3	98.4 ± 3.3	88.5 ± 2.1	101.3 ± 1.9
DBP	Tap	*y* = 19974.01*x* − 2334.41	0.999	0.2	0.5	2.6	1.4	101.4 ± 5.0	96.7 ± 0.5	98.6 ± 1.6
	River	*y* = 20602.78*x* + 4967.80	0.998	0.2	0.5	1.2	5.1	96.1 ± 6.4	101.2 ± 1.9	102.5 ± 0.7
	Lake	*y* = 20508.70*x* + 6984.56	0.998	0.2	0.5	0.3	1.1	95.7 ± 3.9	99.7 ± 4.8	100.4 ± 3.2
	Sea	*y* = 19648.01*x* + 4635.30	0.999	0.2	0.5	4.8	8.8	97.1 ± 0.8	104.6 ± 3.1	101.9 ± 2.0
DPeP	Tap	*y* = 18080.12*x* − 4170.32	0.999	0.2	0.5	1.9	7.2	100.3 ± 6.1	96.6 ± 0.9	97.9 ± 1.6
	River	*y* = 18603.57*x* + 3207.69	0.999	0.2	0.5	1.1	5.4	80.9 ± 5.3	101.3 ± 1.2	101.2 ± 0.8
	Lake	*y* = 18494.86*x* + 4973.91	0.998	0.2	0.5	0.8	2.6	85.8 ± 3.5	101.6 ± 6.0	99.2 ± 3.2
	Sea	*y* = 17767.28*x* + 2667.39	0.999	0.2	0.5	4.0	9.0	80.8 ± 1.3	106.3 ± 2.7	100.6 ± 1.9

**Table 2:** The results of five green evaluation methodologies.

AES		GAPI	AGREE	AGREEprep	SPMS
	PPs	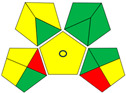	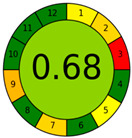	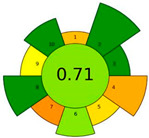	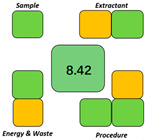
1. Reagents	6				
1.1 Acetonitrile	4				
1.2 Heptanoic acid	1				
1.3 Sodium acetate	1				
2. Instruments-HPLC	0				
3. Waste	9				
Total PPs	15				
Eco-scale score	85				

**Table 3:** Comparisons of ELLME with other liquid phase extraction methods for chromatography analysis of PAEs.

PAE	Environmental Sample	Pretreatment Method	Extraction Reagent	Extraction and Phase Separation Device	Detection Instrument	LOQ (μg L^−1^)	LOD (μg L^−1^)	EF	ER (%)	SPMS Score	Reference
Dibutyl phthalate Benzyl butyl phthalate	River water Lake water	LLE	Tetrabutylammonium iodide + Decanoic acid (3000 μL)	Ultrasonic bath (10 min) Water bath (70 °C)	HPLC-UV	1.0–1.6	0.3–0.9	-	90–98	6.74	[31]
Diethyl phthalate Dibutyl phthalate Dioctyl phthalate Diethylhexyl phthalate	Tap water	HLLME	Dibutylamine (48 μL) Acetic acid (10 μL) Sodium carbonate (250 mg)	Agitator (0.5 min)	GC-FID	11.8–41.3	3.6–12.5	196–394	39–78	7.26	[32]
Dimethyl phthalate Diethyl phthalate Dibutyl phthalate Dipentyl phthalate	Tap water	DLLME	Cresol (200 mg) Butyl lactate (20 μL)	Vortex mixer (1.8 min) Ultrasonic bath (8 min) Centrifuge (8 min)	GC-FID	1–4	0.5–2	256–467	81.8–113.1	5.68	[33]
Didecyl phthalate	Sea water	DLLME	Octanol (500 μL) Acetonitrile (400 μL)	Agitator (20 min) Centrifuge (5 min)	HPLC-DAD	59	20	25	84.5–93.6	5.58	[34]
Dimethyl phthalate Diethyl phthalate Dipropyl phthalate Dibutyl phthalate Dipentyl phthalate	Tap water River water Lake water Sea water	ELLME	Heptanoic acid (200 μL) Sodium acetate (75 mg)	/	HPLC-UV	0.5	0.2	35	80.2–106.3	8.42	This Study

HLLME: homogeneous liquid–liquid microextraction. DLLME: dispersive liquid–liquid microextraction. GC-FID: gas chromatography–flame ionization detection.

## Data Availability

The data can be found in Supplementary Materials.

## Supplementary Materials

The following supporting information can be downloaded at: https://www.mdpi.com/article/10.3390/molecules29245908/s1. Figure S1: Chromatograms of the water samples. (A) PAE standards at a concentration of 5 μg L⁻¹; (B) tap water; (C) river water; (D) lake water; (E) sea water. 1. Dimethyl phthalate (DMP), 2. diethyl phthalate (DEP), 3. dipropyl phthalate (DPrP), 4. dibutyl phthalate (DBP), and 5. dipentyl phthalate (DPeP). Figure S2: The sample preparation metric of sustainability (SPMS) scores for reported methods. (A) literature [31]; (B) literature [32]; (C) literature [33]; (D) literature [34]. Table S1: Preparation of four environmental water samples. Table S2: Greenness assessment of the ELLME-HPLC technique using AES. Table S3: Greenness assessment of the ELLME-HPLC technique using AGREEprep.
