# Supplementary material for: Emulsive Liquid–Liquid Microextraction for the Determination of Phthalic Acid Esters in Environmental Water Samples
Source: Molecules. 2024 Dec 14;29(24):5908. doi: 10.3390/molecules29245908 (PMC11679983; doi:10.3390/molecules29245908)
Supplement: Supplementary file 1 [file molecules-29-05908-s001.zip › molecules-3343256-supplementary.pdf]

Supplementary Material:

**Emulsive liquid-liquid microextraction for the determination of phthalic acid esters in environmental water samples**

Xinyuan Bi[a,b,c], Chi Zhang[a,b], Xiaorong Xue[a,b], Shangjun Su[a,b], Zhiping Yang[a,b], Xu Jing[d], Qiang Zhang[a,b*]

[a] *College of Resources and Environment, Shanxi Agricultural University, Taigu, Shanxi 030801, China*

[b] *Institute of Eco-environment and Industrial Technology, Shanxi Agricultural University, Taiyuan, Shanxi 030031, China*

[c] *College of Agricultural Economics and Management, Shanxi Agricultural University, Taiyuan, Shanxi 030006, China*

[d] *College of Food Science and Engineering, Shanxi Agricultural University, Taigu, Shanxi 030801, China*

*Corresponding authors:

Zhang Qiang, College of Resources and Environment, Shanxi Agricultural University, Taigu, Shanxi 030801, China; E-mail: zhangqiang0351@163.com.

Number of pages: 6

Number of figures: 2

Number of tables: 3

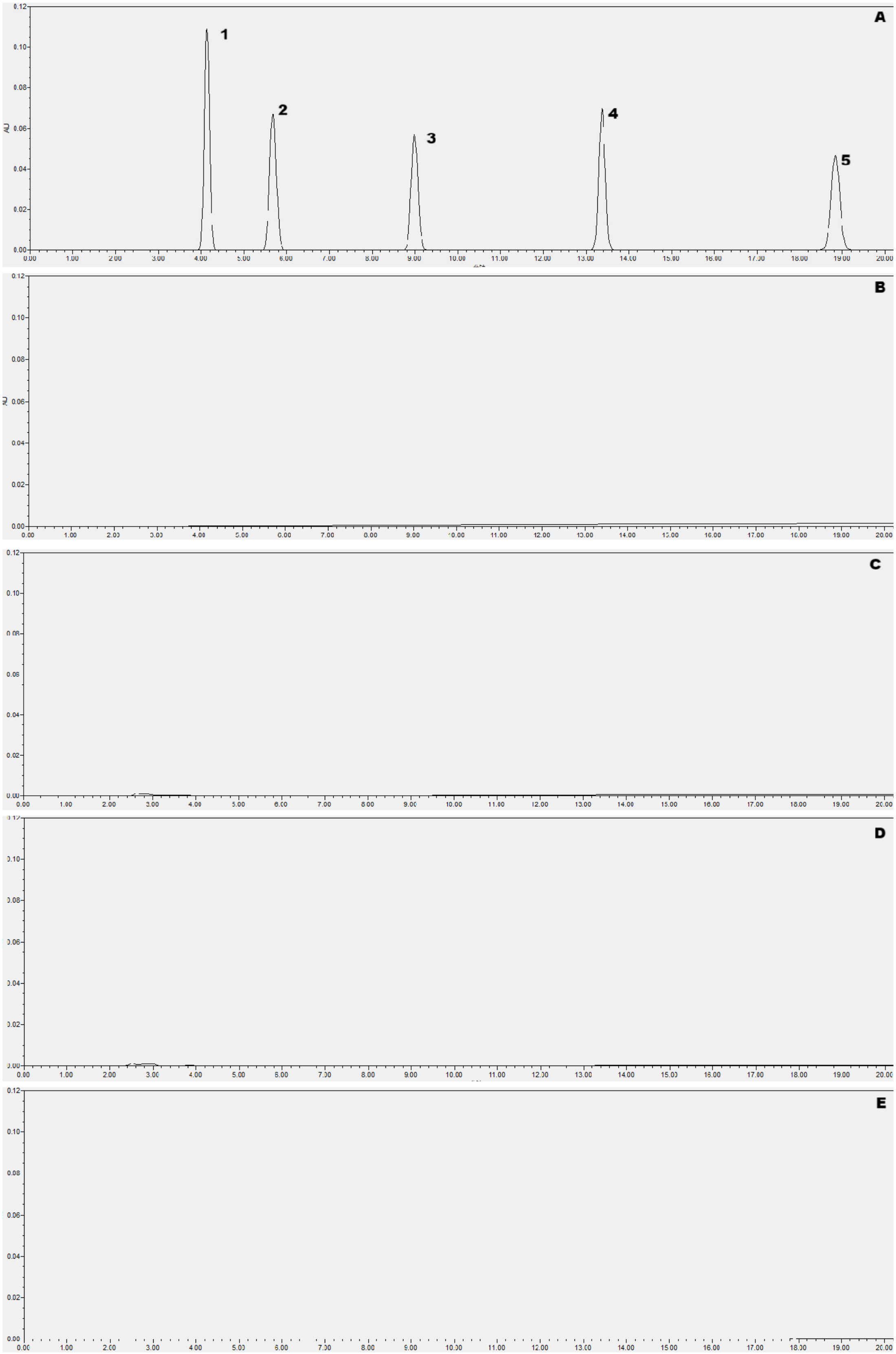


Figure S1: Chromatograms of the water samples. (A) PAE standards at a concentration of 5 µg $L^{-1}$; (B) tap water; (C) river water; (D) lake water; (E) sea water. 1. Dimethyl phthalate (DMP), 2. diethyl phthalate (DEP), 3. dipropyl phthalate (DPrP), 4. dibutyl phthalate (DBP), and 5. dipentyl phthalate (DPeP).

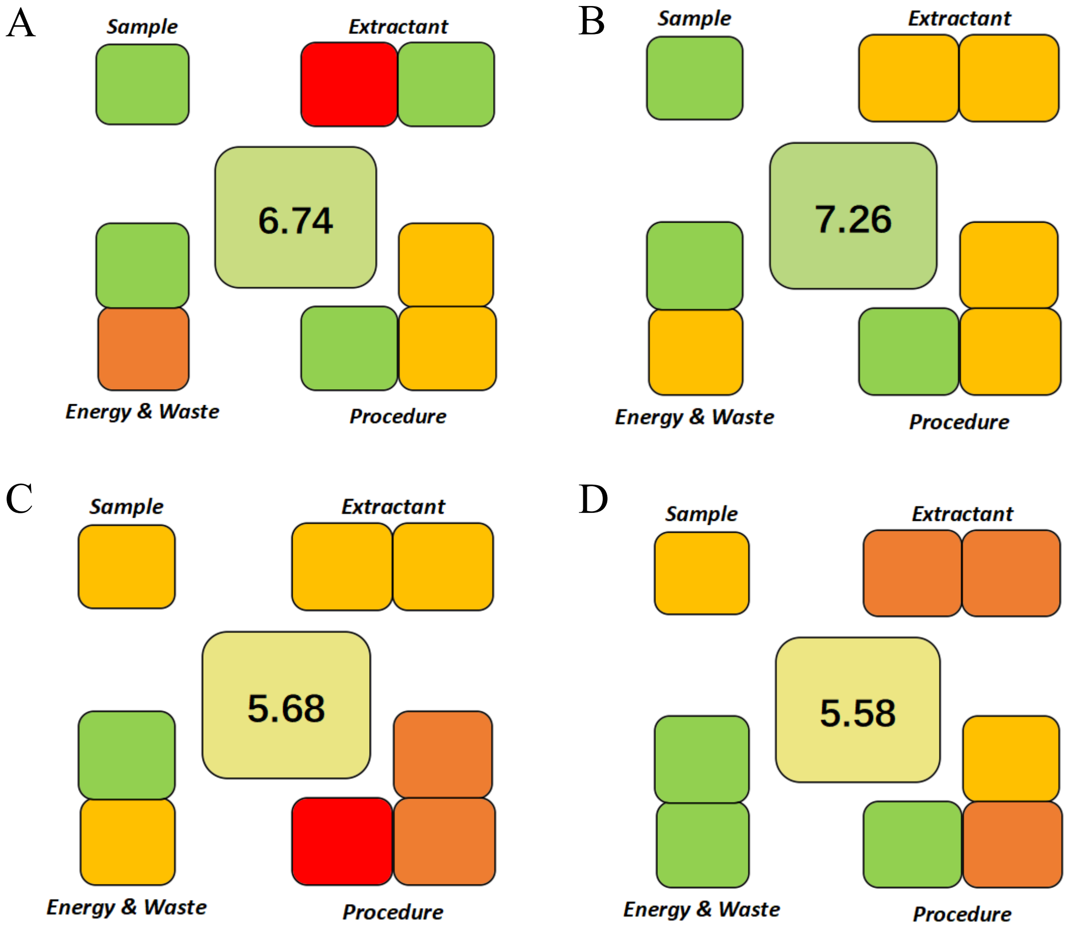


Figure S2: The sample preparation metric of sustainability (SPMS) scores for reported methods. (A) literature [31]; (B) literature [32]; (C) literature [33]; (D) literature [34].

**Table S1** Preparation of four environmental water samples.

| Sample | Coordinates | Storage conditions | Number of samples | pH | Filter | Date |
|---|---|---|---|---|---|---|
| Tap water | Taiyuan, 112.58 37.77 | Glass bottles RT | 15 | 7.94 | ANPEL, organic syringe filter, 0.22-μm | 2024.09.13 |
| River water | Taiyuan, 112.51 37.91 | Glass bottles RT | 15 | 7.72 | ANPEL, organic syringe filter, 0.22-μm | 2024.09.05 |
| Lake water | Taiyuan, 112.50 37.77 | Glass bottles RT | 15 | 7.69 | ANPEL, organic syringe filter, 0.22-μm | 2024.09.08 |
| Sea water | Xiamen, 118.03 24.26 | Glass bottles RT | 15 | 7.43 | ANPEL, organic syringe filter, 0.22-μm | 2024.09.10 |

RT: room temperature

**Table S2** Greenness assessment of the ELLME-HPLC technique using AES.

| | | | Penalty points |
|---|---|---|---|
| 1. Reagents | | | |
| 1.1 Acetonitrile | Amount | <10 mL | 1 |
| | Hazard type | Danger | 2 |
| | Hazard amount | 2 | 2 |
| | | | Total PPs=4 |
| 1.2 Heptanoic acid | Amount | <10 mL | 1 |
| | Hazard type | Warning | 1 |
| | Hazard amount | 1 | 1 |
| | | | Total PPs=1 |
| 1.3 Sodium acetate | Amount | <10 mL | 1 |
| | Hazard type | Warning | 1 |
| | Hazard amount | 1 | 1 |
| | | | Total PPs=1 |
| 2. Instruments | | | |
| 2.1 HPLC | Energy (kW h per sample) | ⩽0.1 | 0 |
| | Occupational hazard | No gas or vapor into air | 0 |
| | | | Total PPs=0 |
| 3. Waste | | | |
| | 3.1. Waste amount (mL) | 1–10 | 3 |
| | 3.2. Waste treatment | No treatment | 3 |
| | | | Total PPs=9 |
| Total penalty points | | | 15 |
| Eco-scale score | | | 100-15=85 |

**Table S3** Greenness assessment of the ELLME-HPLC technique using AGREEprep.

| Criterion | | Score | Weight |
|---|---|---|---|
| 1. Sample preparation placement: | On site | 0.33 | 1 |
| 2. Hazardous materials: | 0 mL | 1 | 5 |
| 3. Sustainability, renewability, and reusability of materials: | Not set | 1 | 2 |
| 4. Waste: | 8 mL | 0.29 | 4 |
| 5. Size economy of the sample: | Mass or volume of the sample: 7 mL | 0.38 | 2 |
| 6. Sample throughput: | 20 samples/h | 0.71 | 3 |
| 7. Integration and automation: | Sample prep. steps: 2 steps or fewer, Manual systems | 0.25 | 2 |
| 8. Energy consumption: | 0 W | 1 | 4 |
| 9. Post-sample preparation configuration for analysis: | GC and HPLC with non-MS detection, atomic absorption spectroscopy, capillary electrophoresis, etc. | 0.5 | 2 |
| 10. Operator's safety: | Not set | 1 | 3 |
